# Eribulin in Triple Negative Metastatic Breast Cancer: Critic Interpretation of Current Evidence and Projection for Future Scenarios

**DOI:** 10.7150/jca.35109

**Published:** 2019-10-12

**Authors:** Laura Pizzuti, Eriseld Krasniqi, Giacomo Barchiesi, Marco Mazzotta, Maddalena Barba, Antonella Amodio, Gioia Massimiani, Fabio Pelle, Ramy Kayal, Enrico Vizza, Antonino Grassadonia, Silverio Tomao, Aldo Venuti, Teresa Gamucci, Paolo Marchetti, Clara Natoli, Giuseppe Sanguineti, Gennaro Ciliberto, Patrizia Vici

**Affiliations:** 1 Division of Medical Oncology 2, IRCCS Regina Elena National Cancer Institute, Via Elio Chianesi, 53, 00144 Rome, Italy.; 2 Department of Clinical and Molecular Medicine, "Sapienza" University of Rome, Azienda Ospedaliera Sant'Andrea, 00189 Rome, Italy.; 3 Department of Surgery, IRCCS Regina Elena National Cancer Institute, Via Elio Chianesi 53, 00144, Rome, Italy.; 4 Radiology and Diagnostic Imaging Department, IRCCS Regina Elena National Cancer Institute, Via Elio Chianesi 53, 00144, Rome, Italy; 5 Department of Experimental Clinical Oncology, Gynecologic Oncology Unit, IRCCS "Regina Elena" National Cancer Institute, Rome, Italy; 6 Department of Medical, Oral & Biotechnological Sciences University G. D'Annunzio, Chieti-Pescara, Italy; 7 Department of Radiological, Oncological and Anatomo-Pathological Sciences, 'Sapienza' University of Rome, Policlinico Umberto I, I-00161 Rome, Italy; 8 HPV-Unit-UOSD Tumor Immunology and Immunotherapy, IRCCS Regina Elena National Cancer Institute, Via Elio Chianesi 53, Rome, Italy.; 9 Medical Oncology, Sandro Pertini Hospital, Rome, Italy.; 10 Department of Radiation Oncology, IRCCS Regina Elena National Cancer Institute, Via Elio Chianesi 53, 00144, Rome, Italy.; 11 Scientific Direction , IRCCS Regina Elena National Cancer Institute , Via Elio Chianesi 53, 00144, Rome, Italy.

**Keywords:** eribulin, triple negative subtype, metastatic breast cancer

## Abstract

Triple negative breast cancer (TNBC) is characterized by distinctive biological features that confer an aggressive clinical behavior. In TNBC patients, the absence of well-defined driver pathways such as hormonal receptor expression or hyperactivation of the human epidermal growth factor receptor 2 (HER2) significantly reduce the spectrum of therapeutic options, which are currently mainly confined to chemotherapy. Thus far, median overall survival for patients with metastatic TNBC is about 9-12 months with conventional cytotoxic agents. However, the heterogeneity recently revealed at a gene expression level inside the TNBC family may help inform therapeutic decisions concerning the use of chemotherapy and hopefully lead the way to novel targeted options that include immunotherapy. Eribulin, a halichondrin class antineoplastic drug, is currently recommended for treatment of HER2 negative metastatic or recurrent breast cancer (BC) previously exposed to anthracyclines and taxanes, also for patients with a TNBC. It is currently indicated from the second line of treatment. In this review, we aim to analyze a wide range of cumulated evidence on eribulin use in TNBC including preclinical studies, intervention and observational clinical trials. Data from the real-world setting and the emerging evidence increasingly substantiating the rationale for combinations with new generation treatment strategies, e.g., PARP-inhibitors, immune checkpoint inhibitors, will be also discussed.

## Introduction

Triple negative breast cancers (TNBCs) are defined as tumors testing negative for estrogen receptors, progesterone receptors and human epidermal growth factor receptor 2 (HER2). These tumors exhibit a particularly aggressive biologic behavior and have a poor prognosis, with chemotherapy still representing the mainstay of treatment in both the early and metastatic settings 1. Within the TNBC group, gene expression profiling has led to the identification of six distinct molecular subtypes, namely, the basal-like BL1 and BL2, immunomodulatory (IM), mesenchymal (M), mesenchymal stem-like (MSL) and luminal androgen receptor (LAR) subtype. For each of these representative subtypes, differences in terms of gene expression signatures may inform decisions on the therapeutic agent/s of choice, as consistently shown by in vitro experiments. Indeed, different clusters of gene expression can be used as readouts of the hyper-activation of distinct “driver” signaling pathways, which effectively translate into better response to different therapeutic agents of the respective cell lines 2,3. Several combination regimens have been tested to improve the clinical outcomes of patients with advanced TNBC tumors. Although combination therapies have resulted into improved response rates (RR) compared with single agents, there was an increased toxicity and no clear benefit was recorded in patient survival 4. In fact, current treatment guidelines recommend the use of sequential single-agent chemotherapy, except in the presence of visceral crisis or rapidly progressing disease 5. In metastatic TNBC tumors, neither specific chemotherapy agents are considered the preferred option, nor an optimal sequence has been established. Concerning the agent/s of choice for first-line treatment, a taxane-based regimen is widely used, often in combination with bevacizumab 6,7, since anthracyclines are usually given in the neo/adjuvant setting, in patients who were not metastatic at diagnosis. However, also nab-paclitaxel, platinum-compounds such as carboplatin, or vinorelbine, gemcitabine, capecitabine-based therapy and anthracycline rechallenge with liposomal formulations represent valid therapeutic options. About the treatment sequence, the most appropriate choice is commonly made on the basis of the previously administered therapy/s and considering a broad range of disease- and patient-related features, which, particularly in the real-world setting, necessarily include co-morbidities. A further driving element in decision making for metastatic TNBC patients is the availability of intervention trials at the institution of reference. Indeed, much of the current efforts of breast cancer (BC) research are oriented towards satisfying the need of significantly expanding the available armamentarium and provide TNBC patients with novel, highly effective treatment options. Among the innovative therapeutic strategies, Poly ADP-ribose polymerase (PARP) inhibitors represent a promising option especially in patients carrying BRCA1 and/or BRCA2 mutation. Several agents of this class are currently being tested for TNBC treatment across the different settings for patients with defects in the DNA repair pathways 8. Meanwhile, a wide range of further molecules acting on additional signaling pathways are being considered, including agents that target the Notch signaling pathway 9,10, the Wnt/β-catenin pathway, and the Hedgehog pathway 11. Moreover, immunotherapy with immune checkpoint inhibitors combined to chemotherapy demonstrated clinical benefit in terms of overall survival (OS) in a recent phase III trial 12.

In this review, we present and discuss evidence from the most relevant and recent studies on the use of eribulin as a single agent and in combination with chemotherapy and/or biologic therapy, in preclinical studies and in TNBC patients, with a specific, though not exclusive, focus on metastatic TNBC.

## Eribulin mechanism of action and clinical studies in advanced breast cancer

Eribulin is a non-taxane synthetic analogue of halichondrin B, a product that is isolated from the marine sponge *Halichondria okadai*. Eribulin inhibits microtubule polymerization without affecting depolymerization, thus causing less toxicity compared to taxanes. This results into apoptosis through an irreversible mitotic block at the G2-M level 13, 14. Among its non-mitotic mechanisms of action, in recent preclinical studies of human BC models, eribulin was shown to alter the tumor microenvironment. Eribulin elicits anti-angiogenetic properties by affecting pericyte-driven angiogenesis 15, increases tumor oxygen saturation and decreases the plasma concentration of TGF-beta1, which restores oxygen flow to the hypoxic regions within the tumor 16. Additionally, in breast cancer models, eribulin decreases migration and invasiveness in vitro as well as metastatic spread in vivo through inhibition of SMAD2 and SMAD3 phosphorylation 17. Moreover, eribulin impacts negatively on invasion, migration and metastatic spread in preclinical studies 18. Recent evidences concerning tumor microenvironment revealed a predictive and prognostic role of the tumor-infiltrating lymphocytes (TILs) in early BC, as well as in TN disease 19,20. In a recent study, patients with advanced TNBC with high TILs levels receiving eribulin showed an improved disease-free survival (DFS) in comparison with patients with low TILs levels, whereas no differences depending on TILs levels were observed in non-TN disease 21. An interesting study was carried out in 22 advanced breast cancer patients. Peripheral blood samples were collected before eribulin administration, and circulating tumor cells (CTCs) were analyzed. Based on the positivity for vimentine and pan-cytokeratin, cancer cells were defined as mesenchymal or epithelial, respectively. Overall, the number of CTCs increased during disease progression under eribulin treatment. Median progression free survival (PFS) was 14.6 weeks, and significantly shorter PFS was observed in patients with higher levels of total and mesenchymal CTCs at baseline (p=0.0013 and 0.013, respectively). A small number of basal CTCs seemed to be related to longer PFS. This study suggests that CTCs quantization and characterization may be predictive of eribulin efficacy 22.

Data from registrative and clinical trials showed that eribulin has a manageable tolerability profile, mainly consisting of neutropenia, alopecia, fatigue, and peripheral neuropathy 23. In November 2010, the Food and Drug Administration (FDA) approved eribulin in patients with metastatic BC following administration of at least two regimens with an anthracycline and a taxane, based on data from the EMBRACE trial, which demonstrated a statistically significant improvement in OS for patients treated with eribulin compared to those treated with therapy of physician's choice 23. In this study, the 19% of all cases had a TNBC and eribulin was highly effective, with its use being associated with a 29% decrease in risk of death compared to other treatments.

In the Study 301, advanced BC patients pretreated with anthracyclines and taxanes were randomized to receive eribulin or capecitabine as first, second or third-line. Median OS were 15.9 and 14.5 months, respectively, and no differences were observed in RR and PFS. Toxicity was acceptable in both the arms, so the study did not demonstrate superiority of eribulin over capecitabine 24. However, subgroup analyses evaluated OS in different cohorts, and eribulin use showed longer OS in HER2-negative (15.9 versus 13.5 months), estrogen receptor negative (14.4 versus 10.5 months), TN (14.4 versus 9.4 months) disease, and in patients without visceral disease, with more than two metastatic sites involved, and in those pretreated with anthracyclines and taxanes in the advanced setting. Median PFS was similar across the treatment arms 25. The OS benefit was confirmed when pooling data from these two large studies, i.e., the Study 305 and 301 26. Overall, the pooled analysis included data for 1,864 patients, of whom 31.5% were treated in second-line and 32.7% in third-line. In the intent-to-treat analysis, median OS was 15.2 months with eribulin and 12.8 months in the control arm (p=0.003). In patients with HER2-negative disease, the median OS with eribulin was 15.2 versus 12.3 months (*p*=0.002). For patients with TNBC, the median OS was 12.9 months with eribulin compared with 8.2 months in the control (p=0.006). No statistically significant benefit was observed in HER2-positive disease; for this subgroup, the median OS with eribulin was 13.5 versus 12.2 months. This analysis suggested that eribulin improved OS in the overall population compared to control, specially in patients with HER2-negative disease. In addition, the greatest benefit was observed in the TN subgroup. A subsequent pooled analysis of these two studies was carried out in patients who had received at least one prior chemotherapy 27. Overall, data from 1,644 patients were included and OS was significantly more favorable with eribulin versus control (p=0.01); patients in the eribulin arms also showed longer PFS and greater clinical benefit rate (CBR) (p< 0.05 for both).

Apart from registrative trials, several prospective clinical studies of eribulin efficacy and tolerability in first, second and third-line have been conducted thus far [Table 1]. The Belgian expanded access prospectively collected efficacy and toxicity data of 154 heavily pretreated (a median of four previous chemotherapy lines) patients. The overall RR were 24% and 14% in patients pretreated with both vinorelbine and taxanes, 29% in patients with hormonal receptor positive tumors, whereas it was 21% in TN disease. Median PFS and OS were 3.2 and 11.3 months, respectively. These results substantially confirmed those of the pivotal EMBRACE trial 28. A recent multicenter phase II study, the OnSITE, evaluated eribulin as third-line therapy in anthracyclines and taxanes pretreated patients, confirming the usual safety profile. Median PFS and OS were 4 and 13.6 months, and median OS was associated with baseline lower (<5) circulating tumor cells 29. A phase II single arm study of eribulin from first to third-line in advanced disease was conducted in Japan, enrolling 47 patients, with an overall RR of 17%, a median PFS of 4.9 months, and a median OS of 17.4 months. As expected, first-line eribulin led to higher RR and longer PFS/OS in comparison to third-line treatment. Data on toxicity confirmed those from prior trials 30. As concerns the use of eribulin in less heavily pretreated patients, some small phase 2 trials of eribulin given as first-line treatment for advanced disease showed clinical activity and acceptable tolerability 31. An analysis on activity depending on previous anthracyclines and taxanes confirmed eribulin efficacy regardless of prior adjuvant anthracycline/taxane 32.

The Meribel study evaluated eribulin as first-line treatment for aggressive, taxane-pretreated, HER2-negative advanced BC patients recurring after a short DFS, with a median disease free interval of 15.7 months. Fifty-three patients were enrolled. Median time to progression was 4.1 months, overall RR was 20.8%, and CBR was 26.4%. Safety was consistent with the previous clinical experience. This study confirmed that eribulin might be considered also in patients' subsets with less favorable outcomes from prior therapy 33. A Japanese phase II study of eribulin as first (68.8% of the patients) or second-line was reported and showed in 32 patients an ORR of 43.8%, CBR of 56.3%, with a median PFS of 8.3 months. All tumor subtypes benefited from eribulin treatment and the safety profile was acceptable 34.

## Eribulin combination regimens

A multicentre phase II randomized trial of eribulin plus gemcitabine versus paclitaxel plus gemcitabine as first-line treatment for advanced BC enrolled 118 patients, showing a 6-month PFS rate of 72% for patients in the eribulin/gemcitabine arm and 73% for patients in the paclitaxel/gemcitabine arm. Neither significant differences in OS were observed, nor CBR showed significantly differing values across the study arms. Grade 3/4 neurotoxicity was more common with paclitaxel/gemcitabine 35. A phase I dose-finding study of eribulin/capecitabine (JBCRG-18) was carried out in pretreated advanced BC patients. Eribulin 1.4 mg/m2 days 1 and 8 with capecitabine 1000 mg/m2 BID were the recommended doses, showing manageable toxicity and encouraging clinical activity (1/3 response) 36.

Another phase I study of dose escalation of eribulin and S-1 (an oral fluoropyrimidine) was carried out, enrolling 12 patients in 3 cohorts, and the recommended doses were eribulin 1.4 mg/m2, days1 and 8 d and S-1 65 mg/m2. The most common toxicity was neutropenia, with 25% of febrile neutropenia. Overall, a 41.7% RR was recorded 37.

## Eribulin combination regimens with biological agents

Subgroup analysis of data from clinical trials that used standard chemotherapy with or without antiangiogenetic agents (such as bevacizumab or sorafenib) show that the addition of these biological agents yields significant PFS and response benefit, particularly in TNBC patients 38, 39. Unfortunately, randomized studies of bevacizumab failed to demonstrate improvement in OS 40, 41. However, it is possible that a subgroup of TNBC may benefit from antiangiogenetic therapy, but there is lack of predictive markers. As concerns eribulin use combined with other antiagiogenetic agents, a phase II randomized trial evaluated eribulin versus eribulin plus ramucirumab in patients previously treated with anthracyclines and taxanes as third-line or beyond. The study was conducted on the basis of a strong biological rationale in using agents targeting VEGF since this, and more generally, angiogenesis is involved in many key processes regulating carcinogenesis as findings from early pre-clinical models suggested. One hundred forty-one patients were randomized, stratified by previous antiangiogenetic treatments and TN status. Median PFS for the combination arm was 4.4 months and 4.1 months for the eribulin arm (p = 0.35). Median OS for ramucirumab-eribulin arm was 13.5 months compared to 11.5 months in the eribulin arm (p 0.68). Response rate was 21% and 28%, respectively, and toxicity was higher in the ramucirumab arm. The study did not show advantage in clinical outcomes from the combination over eribulin as single agent 42.

An intriguing recent phase I study evaluated the combination of eribulin and balixafortide, a selective antagonist of C-X-C chemokine receptor type 4 (CXCR4). In preclinical studies, this combination showed enhanced cytotoxicity and inhibition of metastases in murine models of TNBC 43. The study enrolled patients with HER2-negative advanced BC, tumor cell CXCR4 expression, previously treated with one to three regimens and at least one endocrine therapy for advanced disease. In the part 2 of the study, these patients received eribulin at standard dose on days 2 and 9 of a 21-day cycle, and balixafortide by e.v. infusion starting from 2mg/kg with dose increments, on days 1-3 and 8-10. Fifty-six patients were enrolled, the maximum tolerated dose was not reached, and the dose chosen was balixafortide 5.5mg/kg on the days above indicated, in combination with eribulin. Overall, RR was 30%, and median PFS was 6.2 months in the expanded cohort. Toxicity was mainly fatigue, neutropenia, infusion-reactions, alopecia, constipation and nausea. Serious adverse events were observed: in details, febrile neutropenia in 9%, pneumonia, constipation and neutropenia in 4%, urinary infections in 5% of the patients. There were two toxic deaths (5%). Preliminary activity of the combination seems encouraging in HER2-negative advanced breast cancer, and warrants further exploration in randomized studies 44.

## Eribulin in the real-world setting

A multicenter observational retrospective Italian study was carried out to evaluate eribulin impact in a real-world patients' population of only women. One hundred and thirty-three advanced BC patients pretreated with at least two chemotherapy lines up to more than five lines were enrolled at 11 oncologic centres. A median of 5 cycles of eribulin were administered, and treatment in some cases was very prolonged, up to 15 cycles. The RR was 21.1%, stable disease was recorded in 57 patients (42.8%), and CBR was reported in 38.3% of the patients. Eribulin was more active when administered as third-line and in HER2-negative tumors. In the small subset of TNBC (14 patients), RR was 21.4%, and CBR 35.7%. Overall, the median PFS was 4.4 months and the median OS was 14.3 months. Toxicity was manageable, with fatigue being the most common side effect, cumulative dose-related. The results of the study confirm the favorable outcomes and the manageable toxicity even in a heterogeneous breast cancer patient population treated in real-world setting 45. Another multicenter retrospective study evaluated eribulin in advanced and heavily pretreated BC patients in real-life setting, recruited at 10 Italian oncologic centres (TROTTER trial). The study included 22 (19.5%) TNBC patients. Overall, RR was 24%, CBR 35.4%, the median PFS was 3.3 months and the median OS 11.6 months, without correlation with BC subtypes. Toxicity was acceptable 46. The ESEMPIO study retrospectively evaluated eribulin treatment in 574 Italian patients who received eribulin as any line of treatment. No safety concerns were raised in the population enrolled. Median PFS and OS were 3.2 and 10.1 months, respectively. Eribulin efficacy was similar across BC subtypes. The outcomes recorded in this real-life study were comparable to those reported in pivotal trials 47.

Another real-world experience on long-term outcomes of patients treated with eribulin in the US community oncology setting evaluated 152 patients treated as first and subsequent lines, with a RR of 69.9% in early lines and 48.8% in third and subsequent lines. Toxicity was acceptable with no new safety issue. Median OS was 23 months in early lines and 14.7 months in later lines, the latter comparable to those of the EMBRACE and 301 trials 48. An Italian retrospective study on 44 heavily pretreated advanced BC patients showed longer PFS in ER-positive/HER2-negative tumors, and more favorable results have been observed in patients pretreated with first-line paclitaxel/bevacizumab in comparison to other first-line regimens. No significant differences in PFS were observed between 12 TN patients and HER2-positive and/or ER positive patients 49. The French expanded access program retrospectively collected efficacy data from the five French oncologic centres that participated in the program. The median duration of eribulin treatment was 3.3 months in 250 heavily pretreated patients. Only 28 patients had TN disease. Overall, RR was 17.8%, median PFS was 4.6 months and median OS 11.8 months, with patients responders to the last microtubule-inhibiting agents (docetaxel, paclitaxel, vinorelbine) showing longer OS and slightly better PFS 50. The efficacy and tolerability of eribulin in elderly population was evaluated in a retrospective real-world study. Median age of recruited patients was 74 years (range 70-92), and patients had a median of three previous chemotherapy regimens, with only 7 patients having TN disease. The median number of cycles administered was 6. The median PFS was 6 months and the median OS was 28 months, with patients who had hormonal receptor positive tumors and lower ECOG experiencing longer PFS. The most common toxicities were fatigue, neutropenia, peripheral neuropathy. Overall, safety and efficacy of eribulin was similar to previous studies and did not differ by age 51. A real-world experience on 52 TNBC patients treated with eribulin at the Russia Oncology Research Centre showed a RR of 9.6%, stable disease in 46% of the patients and a median PFS of 3 months. Most of the patients had visceral metastases (92%) and eribulin was given as first and second-line in 14 patients, and in subsequent lines in the remaining patients. Tolerability was acceptable, with neutropenia being the most common side effect, and grade 2 fatigue and peripheral neurotoxicity occurring in 7.7% of the patients. Two withdrawals of treatment due to toxicity were recorded 52.

## Eribulin in TN subtype

### Preclinical studies with eribulin

An interesting experience in BC cell lines, both TN and non-TN, and correlation with sensitivity to eribulin in vitro was recently carried out. Cell-viability assays, apoptosis, migration and invasion tests were performed, and quantitative rtPCR was conducted after exposure to eribulin. The drug reduced cell viability in both TN and non-TN cell lines. Moreover, eribulin induced apoptosis and decreased migration and invasion, and genes related to malignant transformation resulted differentially expressed after eribulin exposure. The authors concluded that eribulin showed a strong antiproliferative effect on BC cell lines, even though in this study no significant difference between TN and non-TN cells was demonstrated 53. The combination of the histone deacetylase inhibitor OBP-801 and eribulin acts synergistically in inhibiting the growth and inducing apoptosis in TN breast cancer cells 54. A synergistic action was also reported when combining eribulin and a novel cycline dependent kinase (CDK) 2/9 inhibitor, CYC065, in three distinct TN cell lines, through inhibition of non-canonical SMAD3 phosphorylation at the T179 site in the protein linker region, with an increase of p15 and a decreased c-MYC expression. Moreover, the combination increased AP1 activity and reduced activity of NFkB, SP1, E2F, SMAD3. Data were confirmed in a xenograft model of TNBC, opening a hypothetical future scenario concerning innovative eribulin-based combinations 55. Eribulin and carboplatin or cisplatin were tested in two TN cell lines and activity in combination was seen in vivo in the HCC1806 and MX-1 TN models 56. The combination of eribulin and paclitaxel was tested with both concurrent and sequential administration modalities in four TNBC cell lines (MDA-MB-231, Hs578T, MDA-MB-157, Mx-1), and in a mouse xenograft model derived from MDA-MD-231 cell line. The expression of epithelial-mesenchimal phenotypic markers was analyzed. The simultaneous administration produced a synergistic effect in the first two cell lines, which was not obtained in the last two cell lines. Moreover, pretreatment with one drug enhanced sensitivity to the subsequent drug in the first two cell lines, where eribulin also increased E-cadherin expression and decreased the expression of mesenchimal markers, while paclitaxel increased them. Additionally, eribulin sensitivity was increased when epithelial-mesenchimal transition (EMT) was induced by TGF-beta1; conversely, eribulin sensitivity was reduced by a TGF-beta receptor kinase inhibitor. In a xenograft model, concurrent administration of both drugs significantly inhibited tumor growth compared to single drug administration. The single eribulin administration before paclitaxel decreased vimentin expression and reduced tumor volume in xenograft models. Therefore, the combination of eribulin and paclitaxel might be a viable option for patients with TNBC 57. Eribulin was also tested in combination with bevacizumabin MDA-MB-231 TN cell lines, showing activity of the combination. Eribulin combined with everolimus showed activity in MCF-7 and Mx-1 cell lines, and the mesenchymal-like TN cell lines (Mx-1) appeared to be particularly sensitive to PI3K/mTOR inhibitors 56, 58. Moreover, the combination of eribulin and a dual (PARP/tankyrase) inhibitor (E7449) was tested in a MDA-MB-436 cell lines and in TN xenograft model, showing activity 59. A preclinical study demonstrated that eribulin may contribute to maintain or increase the activity of cytotoxic T lymphocytes (CTLs) and their role against cancer, through reduction of immune suppressive regulatory T cells (Tregs) and M2 tumor macrophages 60. The role of eribulin treatment in immune response modulation was also clinically investigated in a small trial including 52 patients with locally advanced or metastatic breast cancer. Programmed death-ligand 1 (PD-L1) expression turning to negative in some patients and the tendency towards an increase in lymphocyte infiltration rate following eribulin treatment were interpreted in terms of improvement of the tumor immune microenvironment, which was described in association with epithelial- mesenchymal transition (EMT) suppression 61. Considering that the anti-programmed cell death protein 1 (PD-1) pembrolizumab modulates the activity of CTLs via its immune-checkpoint blockade, the combination with eribulin has a well-founded rationale.

### Clinical studies with eribulin in TN breast cancer

A phase I-II study with eribulin and a CSF1 inhibitor, PLX 3397, is ongoing in patients with metastatic BC, with phase the II limited to stage lb-II TN cases (NCT01596751) 62. Another ongoing trial, the phase IB VERITAS study, is currently testing the safety and tolerability of the combination of everolimus and eribulin in TNBC patients through pharmacokinetic and pharmacodynamic assessments 63. The ENHANCE1/KEYNOTE-150 phase Ib/II study, whose results were presented at the 2017 San Antonio Breast Cancer Symposium, reported that the combination of pembrolizumab and eribulin showed a 26.4% of RR in 106 patients with metastatic TN cases. In the open-label study, the RR with the combination for untreated metastatic TNBC patients (n=65) was 29.2%. In pretreated patients (n=41) the RR was 22.0%. Overall, there were 3 complete response (CR) to the combination (2.8%). Eribulin was administered at the standard dose and pembrolizumab at a flat dose of 200 mg every 3 weeks. The median age was 55 years (range, 32-88), the median ECOG PS was 0. About half of patients were PD-L1-positive (45.8%). The median duration of treatment with eribulin was 19 weeks (range, 3-70) and 18 weeks with pembrolizumab (range, 3-72). Patients received a median of 6 cycles. The CBR across all treatment settings was 36.8%. The median duration of response (DOR) was 8.3 months, with 14.3% of the patients experiencing a response lasting for more than 12 months. In the first-line setting, the CBR was 40% and the median DOR was 8.3 months. In the second/third-line, the CBR was 31.7% and the median DOR has not been reached yet. Overall, the median PFS was 4.2 months. In the first-line and second/third-line setting, the median PFS was 4.9 and 4.1 months, respectively. The median OS was 17.7 months (first-line and second/third-line setting, 17.7 and 18.3 months, respectively). Interestingly, clinical activity was observed regardless of PD-L1 expression, but in patients with PD-L1 positive tumor the RR with the combination was 30.6%, whereas it was 22.4% in patients with PD-L1 negative tumors. Two CR were reported in the positive subgroup and 1 was recorded in the negative group. Among the evaluable patients, grade 3 adverse events were reported in 47.7% and grade 4events were experienced by 18.7% of the patients. Dose-reduction was performed in 32% of patients and study withdrawal due to toxicity was necessary for 22.4% of patients. Two of these events were in the phase Ib phase of the study and the remaining 22 were in phase II. There were 7 fatal adverse events, which were treatment-unrelated. The most common grade 3-4 toxicities were neutropenia (30.8%), peripheral neuropathy (9.3%), anemia, fatigue, and hypokaliemia (5.6% each). The most common adverse events of all grades were fatigue (73.8%), peripheral neuropathy (62.6%), nausea (61.7%), alopecia (42.1%), and constipation (39.3%) 64.

Since olaparib, a potent PARP-1, PARP-2 and PARP-3 inhibitor, has shown remarkable efficacy in BRCA mutated breast cancer 65, 66, a phase I/II trial tested the combination of 300 mg of olaparib twice daily and eribulin (at standard dose and schedule) in TN advanced BC patients. The primary endpoint of phase II was RR in the central review. Twenty-four patients were enrolled in the phase II. The median age was 46 years, the median number of cycles was 5.5 (up to 28 cycles), and the median number of prior chemotherapy regimens was 3 (range: 2 to 6). Sixteen patients (66.7%) had visceral metastasis. Response rate was 29.2%, median PFS 4.2 months and median OS 14.5 months. Germline BRCA mutation was found in 3 patients of the phase I and in 2 patients of the phase II study. Safety was analyzed separately for phase I and II. Significant severe adverse events (≥G3) were leukopenia (87.5%, 83.3%), neutropenia (87.5%, 83.3%), febrile neutropenia (20.8%, 33.3%), anemia (16.7%, 41.7%) and thrombosis (0%, 8.3%), respectively. Olaparib was recently approved by the FDA for HER2-negative pretreated metastatic BC patients with BRCA germline mutation, on the basis of the results of a phase III prospective trial that demonstrated the superiority of this agent with respect to standard chemotherapy 66. The combination of olaparib and eribulin was quite well tolerated and showed a promising efficacy for advanced TNBC patients 67.

## Discussion

There is a compelling need to improve clinical outcomes when treating TNBC, especially in the setting of patients with advanced disease. Treatment continues to rely predominantly on chemotherapy, possibly provided as sequentially administered single agents. However, the perspectives of improving the length and quality of life of this group of patients is inevitably directing the scientific efforts towards the pursuit of new agents, with chemotherapeutical, targeted or mixed mechanisms of action. On a parallel path, the biological heterogeneity of TNBC needs to be uncovered and defined in its genetic and molecular traits. The existing evidence in support of the key role played by the TNBC molecular profile in affecting treatment outcomes was further reinforced by the doubling of the RR when using carboplatin instead of docetaxel in BRCA1/2 mutation carriers in the TNT Study 68. This same mutation spectrum is representative of the distinctive hallmark of patients who obtained significant clinical benefit when treated with biological agents that inhibit PARP enzyme, such as olaparib, in the OlympiAD trial. On this basis, genomic profiling will briefly identify predictive biomarkers which may help select those patients' subgroups that may derive the greatest benefit from the use of these agents. In recent years, the scientific interest surrounding eribulin has grown significantly, particularly in TNBC patients. It is currently classified as a chemotherapeutical agent. Nonetheless, its mechanism of action includes multiple facets and is still under investigation. In this review, we focused on evidence from both the preclinical and clinical settings concerning the efficacy and potential future role of eribulin in advanced TNBC. The main action of eribulin consists in inhibiting microtubule polymerization, with a safety profile which seems more favorable with respect to other agents acting on cell cytoskeleton, such as taxanes and vinca alkaloyds. Interestingly, growing evidence supports that eribulin mechanism of action also encompasses complex modulations of the tumor microenvironment, including a reduction of tumor hypoxia by vascular remodeling, reversion of the EMT and, more broadly, the decrease of the capability of invasion, migration and metastasizing potential. Data do also show that eribulin exposure increases the rate of TILs, which opens new horizons if taken together with the evidence of more pronounced clinical benefits in higher TILs TNBC patients when treated with chemotherapy and/or immune checkpoint inhibitors. When pursuing an approach that seeks to exploit as much as possible the therapeutical potentials of eribulin, an additional aspect in need of consideration concerns the identification of predictors of eribulin effectiveness. In recent studies, CTCs seem to be eligible as possible candidates, and both the count and subtyping may have some relevance.

Eribulin has demonstrated an advantageous safety profile in the clinical setting. Data from EMBRACE and Study 301, showed that eribulin was an efficacious agent to be used for treatment of metastatic BC patients previously treated with taxanes and anthracyclines, especially in the subset of patients carrying a TN subtype. This evidence has substantiated the approval of eribulin, which was initially used from the third line, subsequently in the second- and further lines of treatment.

After the registrative trials, subsequent studies sought to investigate eribulin efficacy and clarify some main issues concerning its use [Table 1], such as identifying the best line of treatment, finding strategies to improve eribulin efficacy, evaluating its efficacy in the real-world population and indeed studying the efficacy of this agent as a function of tumor biology, particularly with regards to the BC subtype, since previous data showed heterogeneous outcomes and a possible higher efficacy in TNBC. The small prospective studies that investigated efficacy in relation to line of treatment gave mostly confirmed results from the registrative randomized clinical trials (RCTs). Eribulin is efficacious in third-line but also as a fourth-line regimen. As it was expected, the rate of clinical benefits is higher in first-line with respect to the third-line, and the efficacy in first-line is independent on prior adjuvant anthracycline/taxane treatments, or the length of DFS following taxane use 30-32.

As far as it concerns the strategies to increase eribulin efficacy, studies show that the most encouraging path seems the combination with agents that can add synergic anti-cancer activity. Combination with chemotherapeutical agents that have an anti-metabolic effect, such as gemcitabine, capecitabine and S-1 was feasible 35-37. However, the combination of eribulin with gemcitabine did not show superiority in terms of PFS rate when compared to the combination of gemcitabine with paclitaxel for patients with advanced BC in first-line setting 35. More interesting combinations for eribulin use have been sought looking at biological agents. Previous studies showed that adding anti-angiogenetic agents such as bevacizumab to standard chemotherapy for the treatment of BC yielded good results especially in TNBC, even though no OS advantage could be demonstrated. There is a strong biologic rationale for the combined use of eribulin with antiangiogenetic agents. The addition of the anti-angiogenetic agent ramucirumab to eribulin as a third-line and beyond regimen for the treatment of advanced BC, did not show any statistically significant advantage 42. However, these data were obtained in a mixed BC population and future studies should address the efficacy of the same combination in a pure cohort of TNBC patients and possibly in earlier lines of treatment. Other targeted molecules are also being developed at the preclinical level specifically for combination with eribulin. In this context, the combination of eribulin plus the CXCR4 selective antagonist balixafortide showed promising results for the treatment of HER2-negative BC patients 44.

In this manuscript, we also reviewed the main real-world data on eribulin use for metastatic BC patients, including TN subgroups. With the major limitations of RCTs being the highly-selected patients' population, real-world data are crucial to reinforce evidence consistency and support the external generalizability of data from RCTs to the real-world setting. There is retrospective data mostly of patients treated in Europe and USA regarding eribulin treatment of advanced BC in the entire spectrum of previous treatment lines. The efficacy of eribulin in the real-world population is higher in earlier lines of treatment, such as first-line of second-line. This is consistent with data from the RCTs. However, this agent preserves its efficacy also in subsequent lines, and even in heavily pretreated patients. The treatment is safe across all lines of treatment and toxicities were shown to be manageable also in the elderly population. Retrospective data show that eribulin has an overall higher activity for HER2-negative BC and very consistent RR in TNBC. Very interestingly, some data also suggest that previous treatment with taxanes not only doesn't affect eribulin activity negatively, but it could also be a predictor of higher efficacy.

Overall, the available evidence advocates a promising role of eribulin in the treatment of TNBC. Studies were conducted specifically in TN subtype BC treated with eribulin. However, preclinical data on TN and non-TN BC cell lines showed a strong anti-proliferative effect of eribulin in both types of cells but no difference was recorded in relation to different subtypes. The activity of eribulin in TNBC could be synergistically increased by combining it with agents having a different mechanism of action, as it has been suggested by other preclinical data. Preclinical studies in TN cell lines tested the combination of eribulin with other chemotherapeutic agents, such as platinum-based agents and taxanes, showing feasibility and promising results in terms of synergistically augmented activity. Studies also investigated combination with bevacizumab or everolimus, and the activity was increased in TN cell lines.

Moreover, combinations of eribulin with newer targeted agents such as OBP-801, CDK2/9 inhibitor and PARP/tankyrase inhibitor in separate studies, showed synergy of action when tested on TN cell lines. Very importantly, some other data showed that eribulin may also have an immune modulating mechanism. Eribulin treatment reduces Tregs and M2 tumor macrophages, could cause PD-L1 to turn negative and could increase TILs, opening new hopes for combination with agents such as immune checkpoint inhibitors. Clinical trials have been already conducted and others are ongoing using eribulin as an anti-neoplastic agent specifically for TNBC subtype. The safeties of eribulin plus everolimus or newer molecules such as CSF-1 inhibitor for the use in metastatic TNBC are being investigated. Based on what was observed in preclinical studies regarding a possible synergy between eribulin and immune modulating agents, a clinical trial investigated the combination of eribulin plus the anti-PD-1 pembrolizumab in metastatic TNBC patients showing a higher CBR of 40% in the first line which decreased, but was still significant, in those patients who received the combination as subsequent line of treatment. The RR was higher in patients that had tumors positive for PD-L1 expression. The treatment was well tolerated. Moreover, following the efficacy results of olaparib in BRCA mutated BC patients, another trial tested the combination of eribulin with olaparib for pretreated metastatic TNBC patients unselected for BRCA mutation. The combination was feasible and yielded a consistent RR which reached the overall value of 29.2%.

In a new era of continuously emerging targeted agents and immunotherapies, eribulin shows to have all the potentials for maintaining a significant role in the therapeutic management of metastatic BC, particularly the TN subtype. Research efforts should be invested in finding new predictors of effectiveness, more effective combinations and the best clinical setting to use eribulin for the treatment of advanced TNBC patiens.

## Conclusions

In summary, identifying new effective therapies for TNBC patients, who have traditionally a poor prognosis, represents an area of highly unmet needs in modern oncology practice. The systemic treatment of TNBC still relies on chemotherapy, but recent genomic advances have revealed distinct TNBC subsets that are sustained by specific transduction pathways, which could be actionable. The BRCA gene mutation is emblematic in this regard, since mutation carriers or patients with BRCAness phenotype obtain better responses from platinum-compounds, and can benefit from specific targeted agents such as PARP inhibitors. There are various new drugs on the horizon for TNBC and, among the new chemotherapeutical agents, eribulin is a highly efficacious and well-tolerated treatment, able to yield an OS benefit, which is a very hard goal to achieve in this setting. Prospective clinical trials and data from real-world practice showed that eribulin yields clinical benefits in any line for the metastatic setting. There are some further hints regarding the possible predictive value of previous taxane treatment as an indicator of higher efficacy. Therefore, predictive factors should be sought not only in the genetics of the tumor, but also in clinical dynamics or other biological factors, such as circulating tumor cells. Eribulin has a multidimensional mechanism of action, which extends from the alteration of cytoskeleton, to the tumor microenvironment modification and immune system modulation, makes it a very versatile agent for being integrated to new treatment strategies. Combinations have been active in the preclinical setting with both chemotherapy and biological agents. Future studies should assess the role of combination with targeted agents such as anti-angiogenetic drugs, mTOR inhibitors, PARP/tankyrase inhibitors, CCXR4 antagonists, CDK2/9 inhibitors, CSF-1 inhibitors etc in the clinical setting. The results obtained so far in large prospective trials are encouraging, as demonstrated by the high efficacy of the combination of eribulin with pembrolizumab or olaparib regardless of BRCA gene status. Future clinical trials should embed the biological heterogeneity of TNBC and seek to identify smaller molecularly identified subsets and the personalization of treatments deserves to be promoted based on genetic and biological markers.

## Figures and Tables

**Table 1:** ** clinical studies with eribulin in metastatic breast cancer patients;** CT=chemotherapy; C= Capecitabine; E= Eribulin; G= Gemcitabine; R= Ramucirumab; P= Paclitaxel; NA= not available; NR= not reached TN= triple negative; TPC= treatment of physician's choice; PFS= progression free survival; OS= overall survival.

Clinical study (r)	Type of study	Number of patients	Number of previous CT lines (median)	Arms	Triple negative (%)	PFS (months)	OS (months)
Cortes J, 2011	Phase III prospective	762	>2	E vs TPC	E 93 (18)	E (all) 3.7 E (TN) NA	E (all) 13.1 E (TN) NA
					TPC 51 (20)	TPC (all) 2.2 TPC (TN) NA	TPC (all) 10.6 TPC (TN) NA
Kaufman PA, 2015	Phase III prospective	1102	≥1	E vs C	E 150 (27.1)	E (all) 4.1 E (TN) NA	E (all) 15.9 E (TN) NA
					C 134 (24.5)	C (all) 4.2 C (TN) NA	C (all) 14.5 C (TN) NA
Aftimos P, 2016	Observational study	154	>3 (4)	E	22 (17)	E (all) 3.2	11.3
						E (TN) NA	NA
Manso L, 2019	Phase II multicentric, single-arm study	59	2	E	12 (20.7)	E (all) 4	13.6
						E (TN) NA	NA
Maeda S, 2017	phase II, multicentric single-arm	47	≤2 (1)	E	NA	E (all) 4.9	17.4
						E (TN) NA	NA
McIntyre K, 2014	phase II, single-arm study	56	0	E	12 (21)	E (all) 6.8	NA
						E (TN) 3.4	NA
Ortega V, 2018	phase II, multicenter single-arm study	53	0	E	24 (45.3)	E (all) 4.1	NA
						E (TN) 3.9	NA
Park YH, 2017	phase II, multicenter study	118	0	E+G vs P+G	E+G 14 (23.7)	E+G (all) 9.6 E+G (TN) NA	E+G (all) 21.3 E+G (TN) NA
					P+G 13 (22)	P+G(all)12.3 P+G (TN) NA	P+G (all) NR P+G (TN) NA
Yardley DA, 2016	phase II, multicenter study	141	>2 (NA)	E+R vs E	E+R 22 (31)	E+R (all) 4.4 E+R (TN)	E+R (all) 13.5 E+R (TN)
					E 21(30)	E (all) 4.1 E (TN) NA	E (all) 11.5 E (TN) NA
Gamucci T, 2014	Retrospective, multicenter	133	≥2 (2)	E	14 (10.5)	E (all) 4.4	14.3
						E (TN) NA	NA
Barni S, 2018	Retrospective, multicenter	574	Any (3)	E	70 (13.3)	E (all)3.2	E (all) 10.1
						E (TN) 2.8	E (TN) 9.1
Rossi S, 2017	Retrospective, single centre	44	≥3 (NA)	E	12 (27.0)	E (all) 2.33	E (all) NA
						E (TN) 1.4	E (TN) NA
Sabatier R, 2018	Retrospective, multicenter	250	≥3 (NA)	E	28 (16.8)	E (all) 4.6	E (all) 11.8
						E (TN) NA	E (TN) NA
De Nonneville A, 2018	Retrospective	60	≥3 (3)	E	7 (12)	E (all) 6	E (all) 28
						E (TN) NA	E (TN) NA
